# Case Report: Atrial fibrillation as the initial presentation of an adult anomalous origin of the left coronary artery from the pulmonary artery

**DOI:** 10.3389/fcvm.2026.1880512

**Published:** 2026-08-26

**Authors:** Cai He, Jun Ma, Wei Wang

**Affiliations:** Department of Cardiology, Wuhan Asia Heart Hospital, Wuhan, China

**Keywords:** ALCAPA, anomalous coronary artery, atrial fibrillation, ischemic cardiomyopathy, left coronary artery transplantation

## Abstract

Anomalous origin of the left coronary artery from the pulmonary artery (ALCAPA) is a rare congenital coronary anomaly characterized by diverse clinical presentations. Atrial fibrillation (AF) as the initial presentation is exceptionally rare and often results in misdiagnosis. This report describes a rare case of adult-type ALCAPA manifesting initially as AF, detailing a comprehensive surgical strategy and long-term outcomes.

## Introduction

Anomalous origin of the left coronary artery from the pulmonary artery (ALCAPA), also known as Bland–White–Garland syndrome, is a rare congenital coronary anomaly, occurring in approximately 1 in 300,000 live births and accounting for 0.25%–0.5% of all congenital heart defects (1–3). The clinical presentation and classification (divided into infantile type and adult type) are primarily dictated by the adequacy of collateral circulation between the right coronary artery (RCA) and the left coronary artery (LCA). In the infantile type, the paucity of collateral vessels precipitates severe myocardial ischemia early in life. Without timely surgical treatment, 90% of affected infants die within the first year (4, 5). Conversely, the adult type is characterized by abundant collateral circulation, permitting survival into adulthood. Herein, we report an unusual case of adult-type ALCAPA presenting primarily with atrial fibrillation (AF). The patient underwent a successful comprehensive surgical strategy and demonstrated a favorable long-term outcome.

## Case report

A 56-year-old previously healthy man presented with a 1-month history of intermittent exertional palpitations. He reported no pertinent family history. Initial vital signs were a blood pressure of 100/64 mmHg, heart rate of 70 beats per minute, and oxygen saturation of 98% on room air. A physical examination revealed a laterally displaced apical impulse, an absolutely irregular cardiac rhythm, and a variable first heart sound intensity; jugular venous pressure was normal, lung fields were clear to auscultation, and no pathologic murmurs were appreciated.

**Figure 1:**
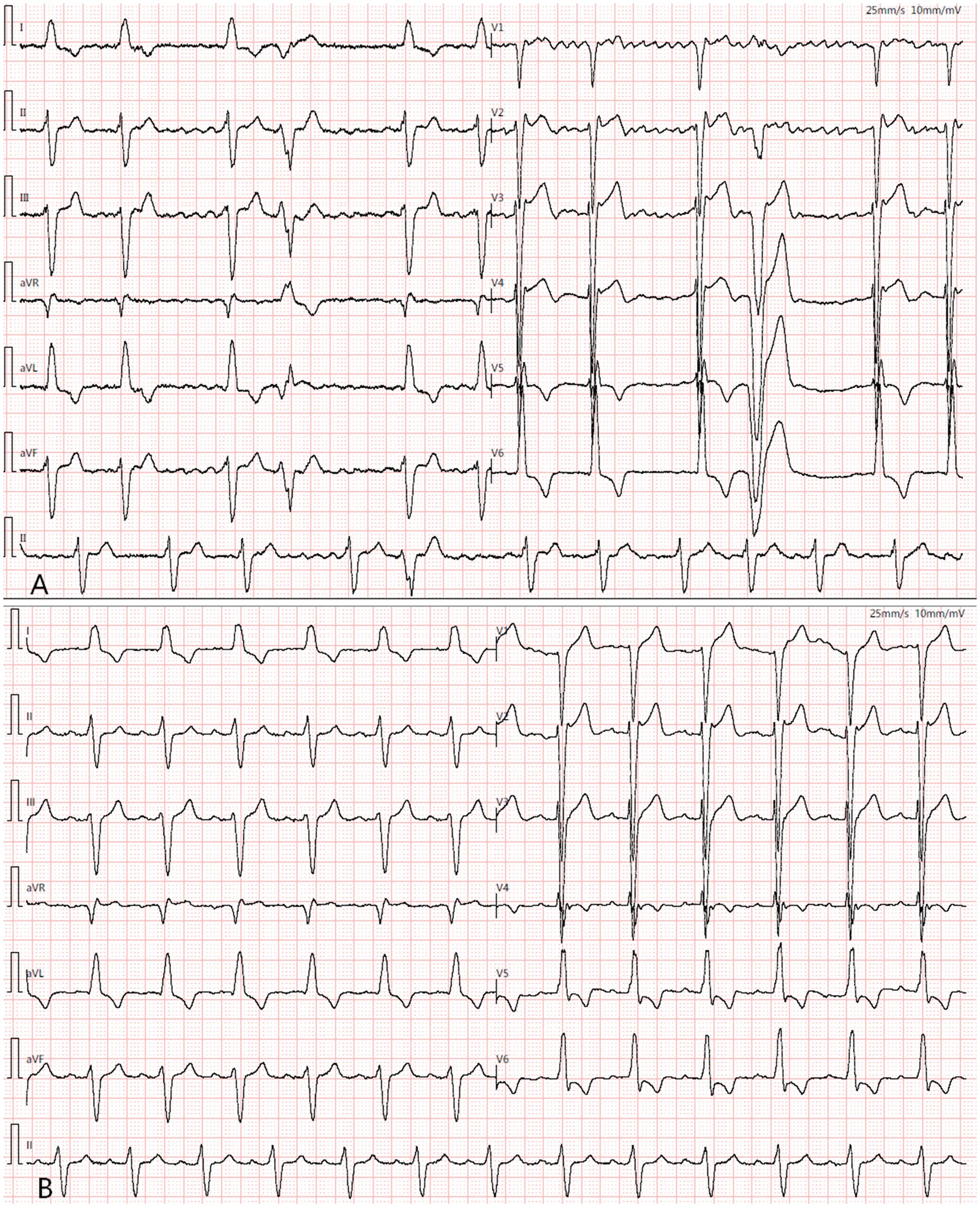
Electrocardiogram. **(A)** At admission, AF, ventricular premature beats, left bundle branch block (The duration of the QRS complex is prolonged by 171 ms. Leads V1-2 show a QS/rS morphology; Leads V5-6 show an R wave.), ST-T changes (ST—segment elevation: 0.1–0.3 mV in leads V1–4; T-wave inversion: in leads I, aVL, and V5-6.). **(B)** After the operation, the sinus rhythm and the left bundle branch block.

Electrocardiography (ECG) demonstrated AF, ventricular premature beats, left bundle branch block, and ST-T changes (Figure 1A). Transthoracic echocardiography (TTE) revealed significant cardiomegaly, with a left atrial anteroposterior diameter of 52 mm and a left ventricular end-diastolic dimension (LVEDd) of 65 mm. Global hypokinesis of the interventricular septum and left ventricular (LV) walls was observed, with a calculated left ventricular ejection fraction (LVEF) of 46%. Severe mitral regurgitation (MR) and moderate tricuspid regurgitation were also noted.

**Figure 2:**
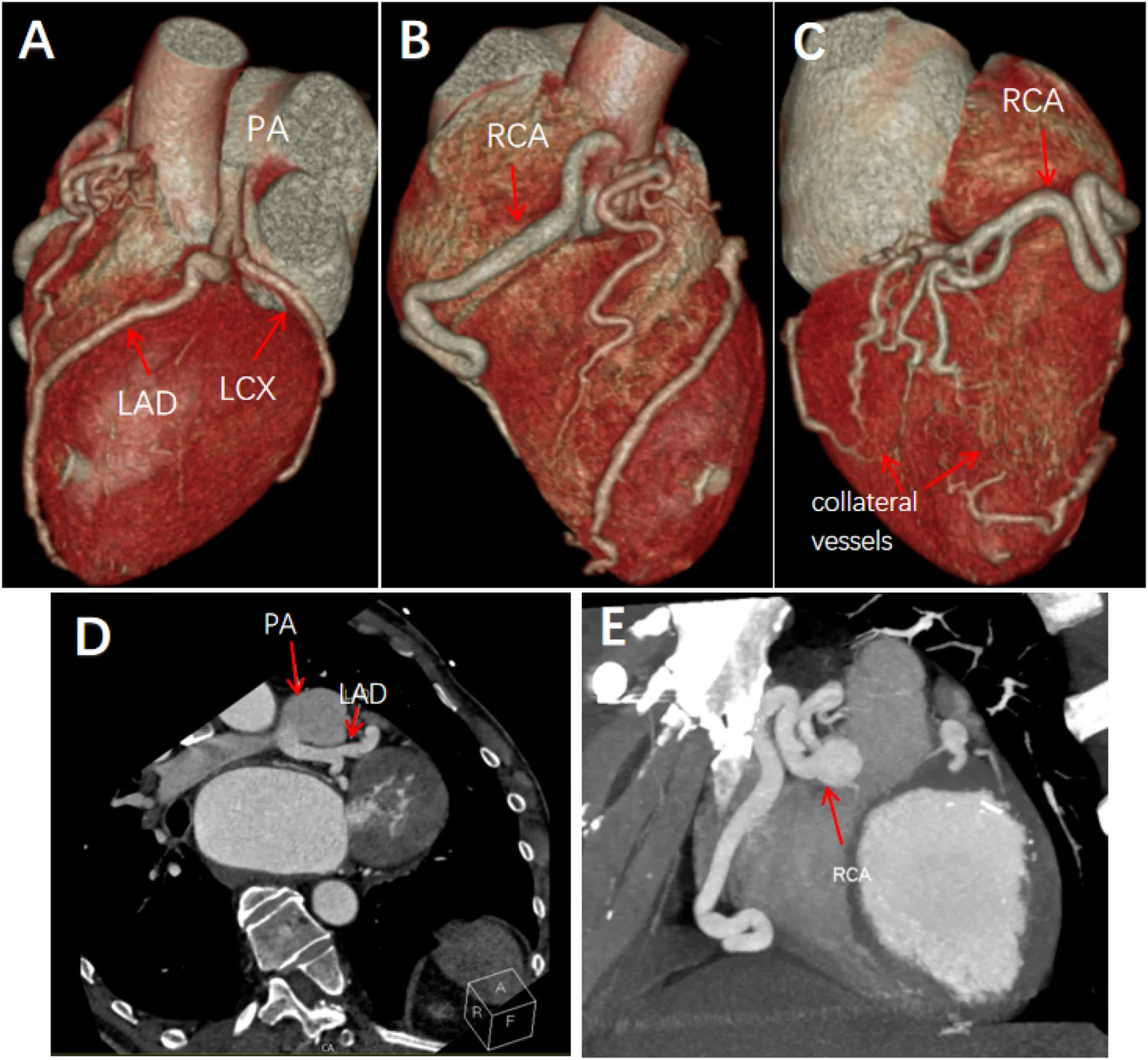
Volume-rendering (VR) images and slice views of coronary computed tomography angiography. **(A)** VR shows that the LCA originates from the PA. **(B)** VR shows that the RCA is tortuous and dilated. **(C)** VR shows multiple small tortuous collateral vessels formed between the LAD and the RCA. **(D)** An oblique sagittal view shows that the left coronary artery abnormally originates from the pulmonary artery. **(E)** A short-axis view shows that the RCA is tortuous and dilated. LCA, left coronary artery; PA, pulmonary artery; RCA, right coronary artery; LAD, left anterior descending branch; LCX, left circumflex branch.

The patient was provisionally diagnosed with atrial fibrillation. In the context of ST-T segment changes, LV dilation, and impaired systolic function, an ischemic cardiomyopathy was suspected. To elucidate the underlying etiology, coronary computed tomography angiography (CCTA) and cardiac magnetic resonance imaging (CMR) were performed. CCTA revealed no atherosclerotic plaques or luminal stenoses. Notably, the LCA arose anomalously from the pulmonary artery. Both the LCA and RCA exhibited marked tortuosity and ectasia, with numerous diminutive, serpiginous collateral vessels bridging the left anterior descending artery (LAD) and RCA territories (Figure 2). CMR revealed extensive subendocardial late gadolinium enhancement consistent with chronic infarction in the septal, anterior, lateral, and apical LV walls (mostly 25%–50% transmurality). Non-viable myocardial mass was 28.7 g (20% of LV mass), with concomitant LV dilatation, systolic dysfunction, and elevated global extracellular volume fraction (38%–41%). Given a CHA₂DS₂-VA score of 2, anticoagulation with subcutaneous enoxaparin (0.4 mL every 12 h) was initiated. Concurrently, medical therapy for heart failure was commenced, including furosemide (20 mg daily), spironolactone (20 mg daily), and metoprolol succinate (23.75 mg daily).

The patient was definitively diagnosed with ALCAPA. Given the unequivocal surgical indications, the patient underwent combined surgical correction. On 26 May 2023, the patient underwent a surgical correction of the anomalous left coronary artery origin with translocation of the left main coronary artery (LCA reimplantation), combined with mechanical mitral valve replacement (MVR), tricuspid annuloplasty, left atrial appendage ligation, and a modified maze procedure via intraoperative cardiac radiofrequency ablation. The surgical technique involved harvesting the LCA with a pulmonary artery button, which was then anastomosed to a punched aperture in the ascending aorta using 5/0 prolene sutures. The pulmonary artery defect was reconstructed with a bovine pericardial patch.

Postoperative recovery was uneventful. By postoperative day 7, ECG showed a sinus rhythm with a left bundle branch block (Figure 1B). TTE revealed normal prosthetic function but LV dysfunction (LVEDd of 65 mm, LVEF of 28%). Medical therapy was optimized accordingly: sacubitril/valsartan (25 mg twice daily), dapagliflozin (5 mg daily), digoxin (62.5 µg daily), and warfarin (3 mg daily, titrated to maintain an international normalized ratio of 2.0–3.0). Other preoperative medications were continued at stable doses. The patient was discharged on 4 June 2023.

The patient was clinically stable at the 1-month follow-up, exhibiting a persistent sinus rhythm without dyspnea or chest tightness. Digoxin and furosemide were therefore withdrawn, with other pharmacotherapies continued unchanged. At the 6-month follow-up, the sinus rhythm was maintained. TTE showed no obvious abnormality in the function of the artificial valve, but the left ventricular systolic function was decreased (LVEDd of 66 mm, LVEF of 34%). A telephonic follow-up through May 2026 indicated NYHA class II status, with occasional exertional chest tightness but no symptoms at rest.

## Discussion

Adult-type ALCAPA is relatively rare, and it often presents initially with heart failure, MR, or sudden cardiac death (6–8). Although arrhythmias are common in ALCAPA, AF is rarely the primary complaint prompting initial medical consultation. The primary novelty of this case lies in the fact that atrial fibrillation was the initial clinical manifestation of ALCAPA. Moreover, the successful implementation of a comprehensive surgical protocol highlights the feasibility of achieving favorable therapeutic outcomes.

The clinical manifestations of ALCAPA range widely from asymptomatic states to severe myocardial ischemia complicated by MR and heart failure (7–9). Severe ischemia may lead to malignant arrhythmias, syncope, and sudden cardiac death. Notably, adult-type ALCAPA often presents with atypical symptoms and frequently coexists with multiple chronic comorbidities, contributing to a substantial early misdiagnosis rate (9–11). Distinct from previous reports, this case uniquely presented with AF as the primary clinical manifestation. The pathogenesis of AF in ALCAPA is likely multifactorial, driven primarily by excessive left atrial volume overload and subsequent structural remodeling. Chronic myocardial ischemia promotes cardiomyocyte apoptosis and extracellular matrix expansion, culminating in atrial fibrosis (12, 13). This fibrotic substrate disrupts the uniformity of myocardial electrical conduction, creating slow-conduction zones that facilitate re-entrant arrhythmias. Furthermore, left ventricular dysfunction and MR augment atrial volume overload, precipitating atrial dilation. This remodeling process further amplifies conduction heterogeneity, fostering the development of multiple micro-re-entrant circuits (14). Concurrently, left heart failure activates the sympathetic nervous system, leading to autonomic dysfunction and altered ion channel kinetics, thereby lowering the threshold for AF initiation.

Once the diagnosis of ALCAPA is confirmed, restoration of a dual coronary circulation is recognized as the standard therapeutic strategy. This can be achieved through multiple surgical approaches, including direct aortic implantation, intrapulmonary tunnel (Takeuchi) procedure, coronary artery bypass grafting, reimplantation of the anomalously originated coronary artery into the aorta, and coronary artery ligation (15–18). The selection of the surgical protocol depends on the ostial location of the LCA, the distance between the aorta and the ostium of the LCA, the stiffness of the tissue structure, and the course of the LCA originating from the pulmonary artery. The direct aortic implantation is considered to be associated with higher technical difficulties and greater risks in adult patients, but it can provide a more physiologically consistent correction effect and achieve better prognosis (17). MR can reflect the severity of left ventricular myocardial ischemia. However, whether MR should be addressed during ALCAPA surgery remains controversial. Some studies suggest that moderate to severe MR associated with structural abnormalities requires concurrent treatment (19, 20). Medical management in ALCAPA serves as an essential adjunct for preoperative stabilization, perioperative hemodynamic support, and chronic postoperative care. The postoperative pharmacologic treatment is directed at promoting reverse remodeling and improving long-term prognosis.

Different from standard simple coronary reimplantation, we formulated a comprehensive strategy targeting the specific pathophysiology of ALCAPA. The resultant MR is largely functional, characterized by ischemic papillary muscle fibrosis and restrictive leaflet motion rather than primary valvular degeneration. Because a simple annuloplasty cannot relieve MR, mechanical MVR was adopted as the definitive solution for sustained valve competence. Furthermore, given the presence of moderate functional tricuspid regurgitation arising from chronic right ventricular volume overload and remodeling, a concomitant tricuspid annuloplasty was performed to preempt progressive right-heart failure. To mitigate the hemodynamic compromise and thromboembolic risks of comorbid AF, we integrated a modified maze procedure and left atrial appendage ligation into the surgical plan. This comprehensive strategy effectively corrected the primary congenital anomaly, while systematically addressing its secondary valvular, electrophysiological, and thrombogenic complications.

A postoperative echocardiography revealed a reduction in the LVEF, consistent with anticipated ventricular unloading following the correction of MR and restoration of the sinus rhythm. Preoperative LVEF assessment in patients with AF and severe MR is notoriously susceptible to overestimation due to three factors: (1) Rhythm variability, where beat-to-beat fluctuations in diastolic filling lead to an underestimation of end-systolic volume; (2) Regurgitant emptying, whereby retrograde flow into the low-pressure left atrium artificially reduces end-systolic volume; and (3) Compensatory hypercontractility, a physiological response to chronic volume overload that masks the underlying myocardial dysfunction (21, 22). Thus, the observed postoperative LVEF decline likely reflects the unmasking of latent systolic impairment rather than new myocardial injury.

## Conclusion

Adult patients with atypical symptoms of ALCAPA are highly prone to misdiagnosis. This case report highlights the importance of including this disease in the differential diagnosis of patients with AF and left ventricular dysfunction. Early management of coronary artery origin abnormalities to resolve myocardial ischemia is crucial for preventing complications such as myocardial infarction, decompensated heart failure, or sudden cardiac death.

## Data Availability

The original contributions presented in the study are included in the article/Supplementary Material, and further inquiries can be directed to the corresponding authors.
